# Evaluation of a commercial packed bed flow hydrogenator for reaction screening, optimization, and synthesis

**DOI:** 10.3762/bjoc.7.132

**Published:** 2011-08-22

**Authors:** Marian C Bryan, David Wernick, Christopher D Hein, James V Petersen, John W Eschelbach, Elizabeth M Doherty

**Affiliations:** 1 Medicinal Chemistry Research Technology, Department of Chemistry Research and Discovery, Amgen, Inc., One Amgen Center Drive, Thousand Oaks, CA 91320, USA; 2 Discovery Analytical Sciences, Department of Chemistry Research and Discovery, Amgen, Inc., One Amgen Center Drive, Thousand Oaks, CA 91320, USA; 3 Research Automation Technology, Department of Chemistry Research and Discovery, Amgen, Inc., One Amgen Center Drive, Thousand Oaks, CA 91320, USA

**Keywords:** catalyst leaching, CatCart^®^, H-Cube^®^, packed bed flow hydrogenation

## Abstract

The performance of the ThalesNano H-Cube^®^, a commercial packed bed flow hydrogenator, was evaluated in the context of small scale reaction screening and optimization. A model reaction, the reduction of styrene to ethylbenzene through a 10% Pd/C catalyst bed, was used to examine performance at various pressure settings, over sequential runs, and with commercial catalyst cartridges. In addition, the consistency of the hydrogen flow was indirectly measured by in-line UV spectroscopy. Finally, system contamination due to catalyst leaching, and the resolution of this issue, is described. The impact of these factors on the run-to-run reproducibility of the H-Cube^®^ reactor for screening and reaction optimization is discussed.

## Introduction

The potential advantages of heterogeneous catalytic flow hydrogenation over traditional batch reactor processes are many and significant [1]. In particular, flow hydrogenation promises strict control of reaction parameters and, therefore, high reproducibility in reaction outcome. Additional advantages include: (1) greater safety due to the containment of the pyrophoric catalyst in a cartridge, column, or microfluidic device; (2) simple product isolation, with no separate catalyst filtration step required; and (3) convenient screening of the reaction conditions and rapid sequential transformations facilitated by automation of the liquid handling.

There have been several reports published on custom flow hydrogenators, such as a Pd-immobilized 200 micron glass channel [2], a simple Pd/C packed bed bubble column reactor [3] and, more recently, a unique glass column packed bed system that introduces hydrogen across a Teflon-AF membrane [4]. In 2004, ThalesNano Inc. [5] was the first manufacturer to commercialize a convenient bench-top hydrogen flow reactor, the H-Cube^®^, designed for smaller-scale use in academic and drug discovery labs [6]. The reactor features a built-in hydrogen generator that functions by the electrolysis of water. Disposable pre-packed catalyst cartridges (CatCart^®^) are also available from ThalesNano. These cartridges consist of a solid catalyst contained within stainless steel tubes fitted with thin 8 micron frits. The manufacturer also demonstrated that high-throughput synthesis can be facilitated on the H-Cube^®^ using a Tecan liquid handler for automated sample injection and collection of the product fractions [7]. Gilson automated liquid handling was similarly added to the H-Cube^®^ by Ley and Ladlow [8], with the system controlled by software custom written at Aitken Scientific [9]. A similar system using a Bhodan robot and Visual Basic software was developed at Abbott Labs [10]. Subsequently, ThalesNano commercialized the system developed by Ley and Ladlow. More recently, ThalesNano has introduced a CatCart Changer^®^ (CCC) attachment with six CatCart^®^ port positions and a column switcher to increase throughput and facilitate screening through multiple catalysts. These ThalesNano H-Cube^®^ systems have been used successfully for a variety of reductions; some of the more notable applications including *O*-debenzylation, CBz-hydrogenolysis, aromatic ring saturation, imine reduction, and enantioselective carbonyl reductions [8,10–13].

**Scheme 1:**
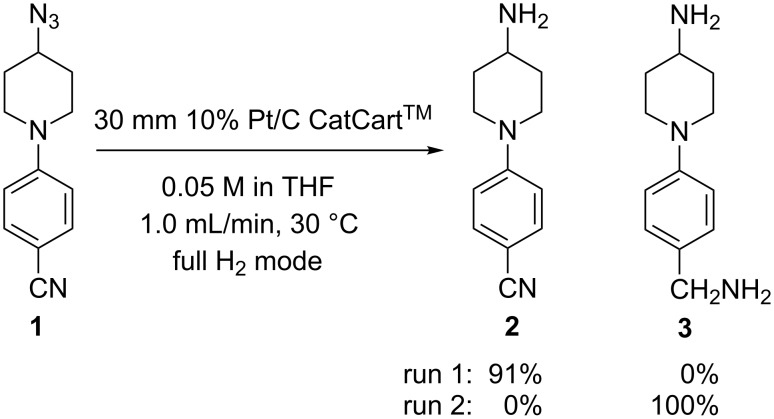
Reduction of 4-(4-azidopiperidin-1-yl)benzonitrile on the H-Cube^®^.

In our hands, the ThalesNano H-Cube^®^ and the H-Cube^®^ with CCC systems had previously produced experimental results that were difficult to explain. For example, we were unable to reproduce the reduction of ethyl pyridine-3-carboxylate by following the conditions reported by Kappe [11], although we were able to achieve similar results under modified conditions [14]. One of the most dramatic results we observed was the complete loss of selectivity in the reduction of an azido group in the presence of an aromatic nitrile (Scheme 1). Initially, the chemoselective reduction of azido nitrile **1** was successfully performed with a 30 mm 10% Pt/C CatCart^®^ to afford amino nitrile **2** in 91% isolated yield. Reproduction of the same conditions on a different occasion, however, resulted in 100% conversion to the fully reduced product **3**. These unexpected results prompted us to seek a better understanding of the parameters affecting reproducibility in flow hydrogenations on the H-Cube^®^.

Surprisingly, to date there have been few reports in the literature characterizing the influence of the H-Cube^®^ system configuration and reaction parameters on the performance and reproducibility. Jones reported consistency in conversion over a series of nitroindole reductions [6] and in the reduction of a library of nitro-group containing molecules through the H-Cube^®^ with the Tecan liquid handler [7]. Ley discussed the effect of temperature on the catalyst activity [8]. Kappe and co-workers showed a correlation between conversion, flow rate, and temperature [11]. But to the best of our knowledge, a systematic investigation identifying the most significant parameters affecting reproducibility and performance of the H-Cube^®^ reactor, particularly during routine use, has not been published.

**Figure 1:**
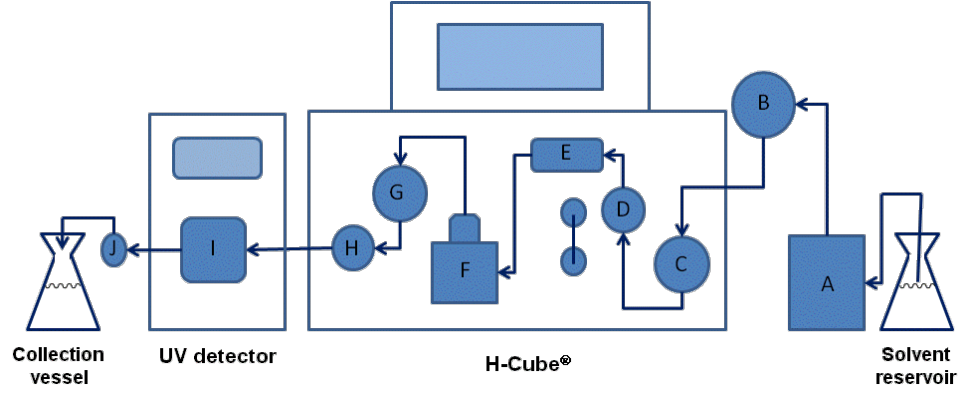
SAH^3^ schematic with injector port and UV detector. Components: (A) pump; (B) six-position manual injector port; (C) inlet pressure sensor; (D) gas–liquid mixing chamber; (E) bubble detector; (F) CatCart^®^ holder; (G) outlet pressure sensor; (H) back pressure regulator; (I) UV flow cell; (J) 10 bar fixed in-line back pressure regulator. Arrows indicate the flow direction.

By incorporating an in-line UV detector, we previously characterized dispersion in the H-Cube^®^ and the effect of that dispersion on the reaction outcome [15]. As a result of that study, we generated a predictive correlation between non-steady state and continuous flow scale-up conditions for simple reductions. In this report, using the reduction of styrene to ethylbenzene over 10% Pd/C as a model, we examine the H-Cube^®^ reactor performance: (1) across different pressure settings, (2) over a series of sequential reactions, and (3) using different commercial CatCart^®^ cartridges. For these studies, two reactor configurations were employed. In the first, a “stand-alone” H-Cube^®^ (SAH^3^) was equipped with a manual injector port and in-line UV detector (Figure 1). The second system (AutoH^3^) consisted of an H-Cube^®^ with CCC, equipped with a Gilson 215 liquid handler and programmed using the ThalesNano auto sampler software.

## Results and Discussion

### Hydrogen variability

The H-Cube^®^ introduces hydrogen to the reactant stream in one of two different modes: “Full H_2_” and “controlled”. In the full H_2_ mode, the maximum amount of hydrogen that can be produced by the electrolytic cell (25 mL/min [16]) is delivered into the gas mixing chamber (D, Figure 1) with no back pressure at the outlet of the system (G, Figure 1). When running in full H_2_ mode, the resultant system pressure is reported on the touch screen panel as 0–1 bar. In comparison to full H_2_ mode, the flow rate of hydrogen in the controlled mode is dependent upon the liquid back pressure. The controlled mode settings (10 bar to 100 bar) are used to set the total back pressure (G, Figure 1) while the system maintains a roughly constant pressure differential between the hydrogen inlet pressure (internal sensor) and the liquid inlet pressure (C, Figure 1). As a consequence of this engineering design, setting the system to the controlled mode introduces less hydrogen into the reactant stream than in the full H_2_ mode setting.

**Figure 2:**
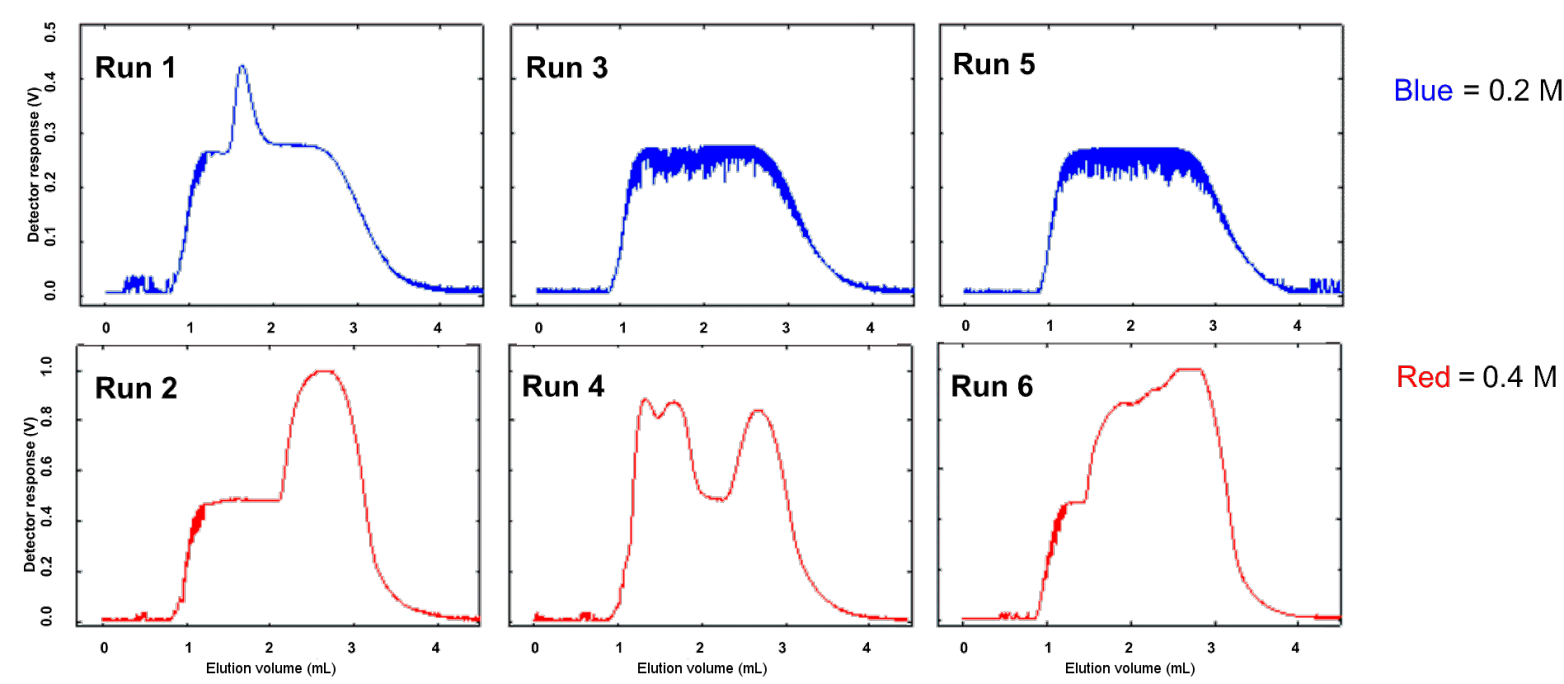
Variation in UV absorbance during the reduction of styrene to ethylbenzene, in the controlled mode on SAH^3^, demonstrating variable hydrogen availability. Reactions were carried out through a 10% Pd/C 30 mm CatCart^®^, at pressure setting 80 bar, MeOH, with flow rate 1.0 mL/min, and temperature 35 °C, and were monitored at 265 nm.

As an indirect measure of the availability of hydrogen during the course of a reaction in the controlled mode setting, the reduction of styrene to ethylbenzene was monitored by in-line UV using the SAH^3^ system. The extinction coefficient of styrene, and the related absorbance at 265 nm, is significantly greater than that of the reduced product ethylbenzene. When the reaction is performed under hydrogen-limited conditions (high substrate concentration, excess catalyst), any increases in absorption observed over the course of the reaction correlate with a decrease in the available hydrogen. Six sequential 2 mL injections of styrene solutions in MeOH, alternating between 0.2 M and 0.4 M concentration, were made and the course of the reductions (80 bar in controlled mode, 1 mL/min, 35 °C) was followed by UV spectroscopy (Figure 2). UV traces from runs 3 and 5 represent uninterrupted, full conversion to ethylbenzene, with short spikes in the curve corresponding to bubbles passing through the flow cell. In four out of six of the reactions (runs 1, 2, 4, and 6), peaks corresponding to unreacted styrene were observed. As expected, the effect of fluctuations in hydrogen availability was more pronounced at higher styrene concentrations (runs 2, 4, and 6). While the system was running, the release of gas from the eluent stream (observed as “sputtering”) would cease intermittently, which was another indicator of fluctuations in hydrogen availability. Whether these fluctuations are due to inconsistent production of hydrogen by the electrolytic cell, or are consequences of the design of the hydrogen flow controlling system, is not clear. This behavior was observed on each of the three unique H-Cube^®^ reactors tested (including an instrument loaned by ThalesNano) and so appears to be associated with the design of the system and is not an isolated mechanical issue.

**Figure 3:**
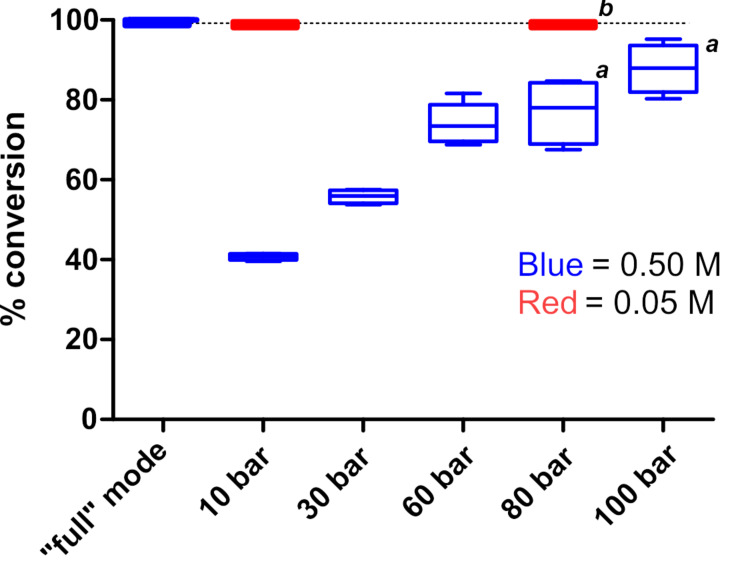
Box plot of data showing the percent conversion of styrene to ethylbenzene as a function of pressure. All reactions were performed using a 10% Pd/C 30 mm CatCart^®^, MeOH, flow rate 1.0 mL/min, at 30 °C, with 1.9 mL per injection and 10 mL product solution collected. The conversion was determined by GC–MS analysis with decane as an internal standard; *n* = 5 unless otherwise indicated. Data represented in blue correspond to 0.50 M styrene in MeOH. Data represented in red correspond to 0.050 M styrene in MeOH. *^a^n* = 4. *^b^n* = 1.

A series of experiments were then performed at different pressure settings with the AutoH^3^ system. In these experiments the full H_2_ mode was compared to the controlled mode at pressure settings of 10, 30, 60, 80 and 100 bar. Final conversion of styrene to ethylbenzene was monitored by GC–MS with decane as an internal standard. The results are shown in Figure 3. At a concentration of 0.5 M styrene, the reactions conducted at 1 bar in the full H_2_ mode afforded the highest average conversion (100%, *n* = 5 experiments). In the controlled mode, the conversion was lower for all settings, with a progressive increase in the conversion correlating with increasing pressure. Significantly, the variability in the conversion increased with increasing pressure, with the largest ranges observed at 60 bar (68.8–81.6%), 80 bar (67.5–84.7%), and 100 bar (80.3–95.2%). This variability was not observed at lower styrene concentrations (0.05 M, Figure 3 results in red), where at both 10 bar and at 80 bar 100% conversion to ethylbenzene was observed. It should be noted that 0.05 to 0.10 M is the working concentration range recommended by the manufacturer. Nevertheless, the results suggest that data must be interpreted with caution when controlled mode settings are used.

In the case of the experiments at 80 bar and 100 bar in Figure 3, several of the reactions were accompanied by a warning in the software results panel: “Collected with Error (instability detected)”. According to ThalesNano, this warning indicates that during some part of the run no hydrogen was detected by the bubble detector (E, Figure 1). The results from these reactions were excluded from statistical analysis, however, the observation prompted us to examine the frequency with which such errors occur during the routine use of the H-Cube^®^. Table 1 shows the accumulated results from 586 experiments recorded in the 13 months after installation of the AutoH^3^ system and software. There were no instabilities detected in any of the 535 experiments run in the full H_2_ mode setting. In comparison, instability was detected in 20% (10 out of 51) of the experiments run in a controlled mode setting. Instability occurred more frequently at higher pressure settings or when a controlled mode experiment came directly after an experimental run at a lower pressure setting (see Supporting Information File 1, Appendix A).

**Table 1:** The number of experiments during which an “instability” was detected and automatically recorded by the AutoH^3^ system. Experiments were conducted at flow rates in the range of 0.5 to 2.0 mL/min.

Pressure setting	Total # experiments	Instability detected

Full H_2_ mode	535	0
Controlled mode 10 bar	10	1
Controlled mode 30 bar	6	0
Controlled mode 60 bar	20	3
Controlled mode 80 bar	10	5
Controlled mode 100 bar	5	1

### Catalyst cartridge variability

**Figure 4:**
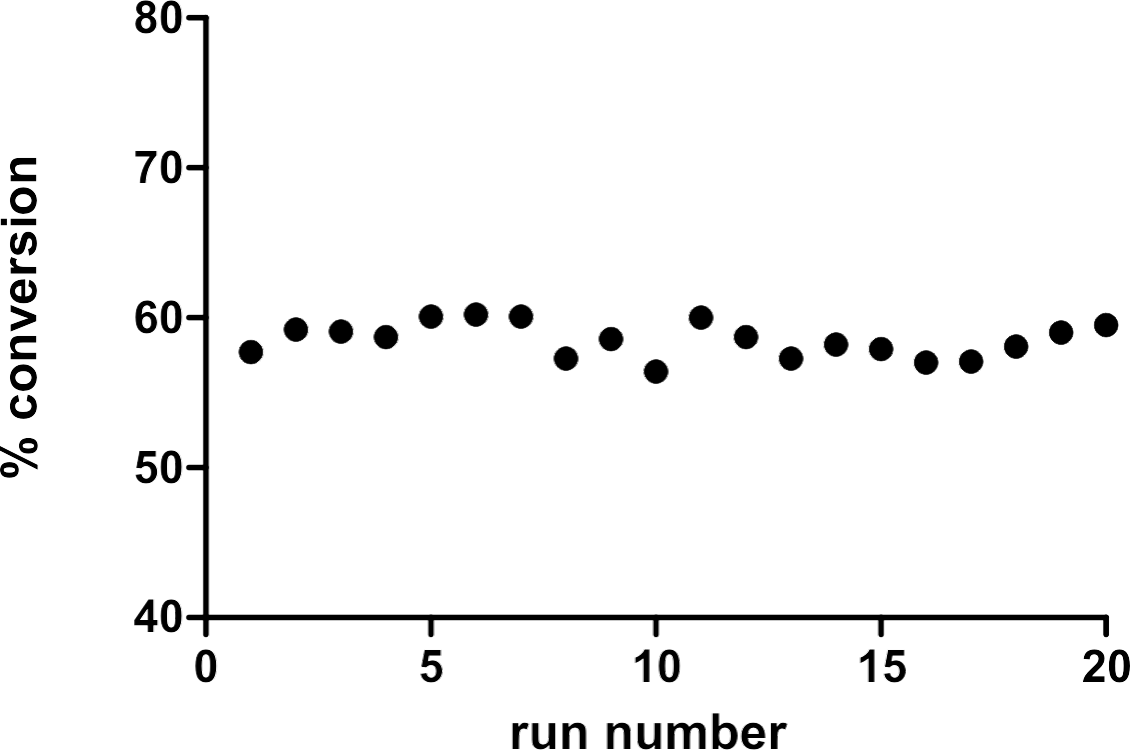
Conversion of styrene to ethylbenzene over 20 reactions in sequence through a single 30 mm 10% Pd/C CatCart^®^. All reactions were carried out with a 10% Pd/C 30 mm CatCart^®^, with 2.0 M in MeOH, at a flow rate of 1.0 mL/min, at 30 °C, with 1.9 mL per injection, and 10 mL product solution collected. The conversion was determined by GC–MS analysis with decane as an internal standard.

The performance of the ThalesNano CatCart^®^ cartridges was examined by measuring the conversion of styrene to ethylbenzene in MeOH over 10% Pd/C. Based on our previous observations, the full H_2_ mode was used to afford the greatest consistency in hydrogen availability. The reactions were run at a high concentration (2.0 M) in order to achieve an incomplete reduction and hence observe any variability in the conversion. The first series of experiments shows the conversion over 20 reactions in sequence through a single CatCart^®^ (Figure 4). The range of variation was low (56.4–60.2%) and the average conversion did not diminish over time, indicating that no activity was lost due to catalyst poisoning or leaching of metal from the catalyst bed.

**Figure 5:**
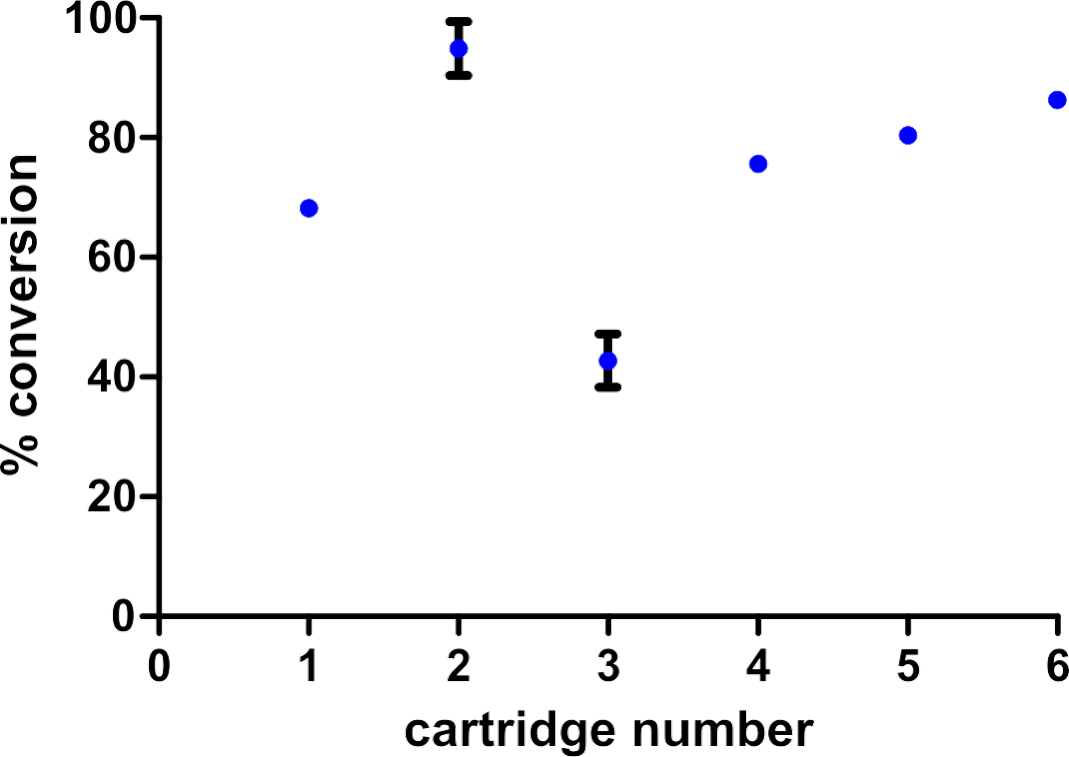
Cartridge-to-cartridge variability of the substrate conversion based on the reduction of styrene to ethylbenzene. All reactions were carried out at 2.0 M in MeOH, at a flow rate of 1.0 mL/min, at 30 °C, with 1.9 mL per injection, and 10 mL product solution collected; *n* = 3 experiments for each cartridge. The conversion was determined by GC–MS analysis with decane as an internal standard. Commercial 10% Pd/C 30 mm CatCart^®^ cartridges were used; numbers 1, 2, and 3 from lot #02946; numbers 4, 5, and 6 from lot #02938.

In the next series of experiments, commercial 30 mm 10 % Pd/C CatCart^®^ cartridges were selected at random from two different lots and were used to examine differences in performance from lot to lot and from cartridge to cartridge. Each cartridge was placed in port position 1 on the AutoH^3^ system, three styrene reductions were performed, and then the cartridge was replaced. In all examples, the catalyst was prereduced under the full H_2_ mode and prewashed with MeOH. The results for six CatCart^®^ cartridges (Figure 5) show a significant variation in the individual cartridge performance (38.5–100% conversion), but with minimal run-to-run variability for any given cartridge over three sequential experiments.

The observed variations in cartridge performance may be attributed to a number of causes such as lot-to-lot variations in the catalyst, variability in mass loading, channeling effects, and column packing technique [17–18]. For example, Makkee and coworkers conducted a thorough investigation of the challenges associated with the scaling down of a “trickle bed” flow hydrogenator [19] and showed that both particle size homogeneity and column packing technique [18,20] have an impact on the conversion and the reproducibility. Whatever the causes, it appears that for any given cartridge the relative loading can be calibrated against a known reaction (such as styrene reduction). As long as that reaction does not reduce the catalytic activity of the cartridge, the subsequent behavior of the cartridge may be predicted with some level of confidence.

### Cross-contamination

**Figure 6:**
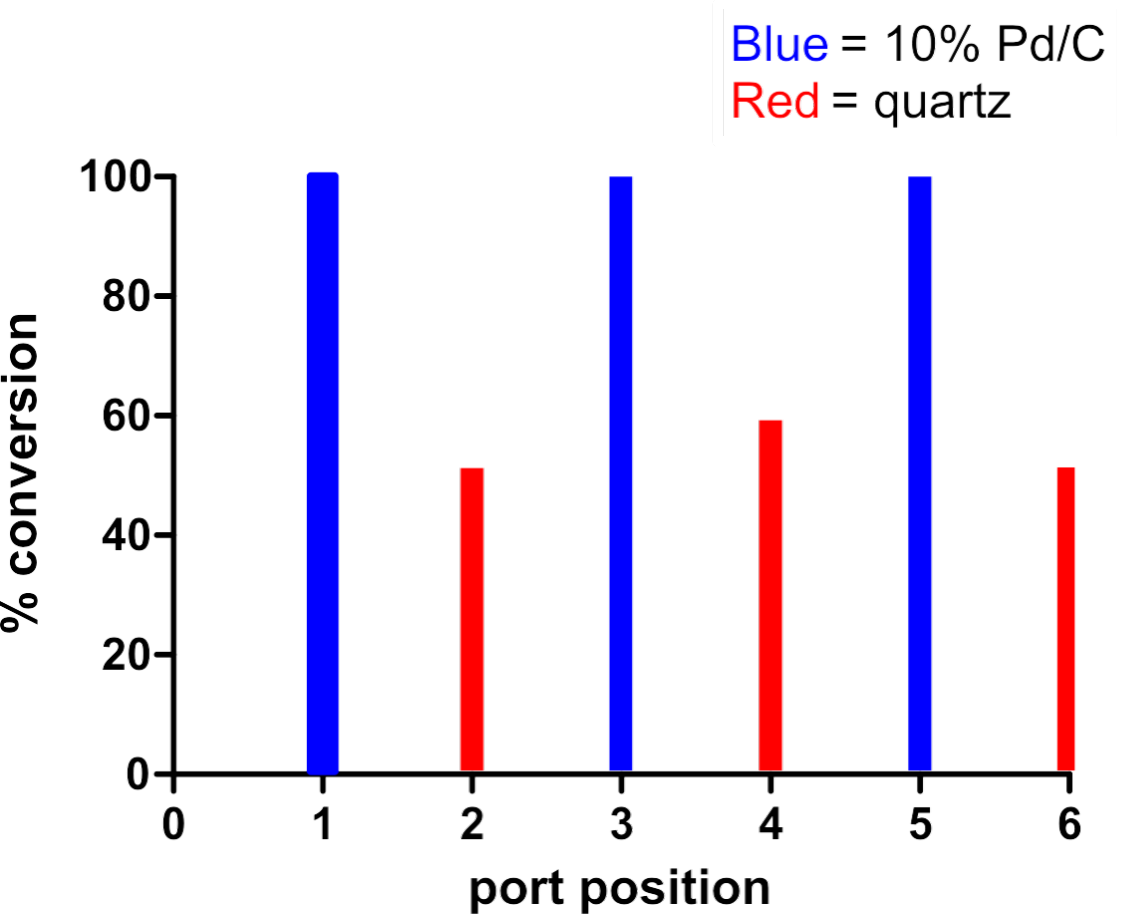
Conversion of styrene to ethylbenzene in a sequence of reactions alternating between 10% Pd/C and quartz-loaded CatCart^®^ cartridges on the AutoH^3^ system. All reactions were carried out at a flow rate of 1.0 mL/min, at 30 °C, with 0.5 M styrene in MeOH, with 1 mL injection per reaction, and run in order from 1 through to 6. The conversion was determined by GC–MS analysis with decane as an internal standard. Data represented in blue correspond to reactions performed through a 30 mm 10% Pd/C CatCart^®^. Data represented in red correspond to reactions performed through 30 mm quartz CatCart^®^.

Catalyst cross-contamination from run to run has a profound effect on the results of the catalyst screening. To investigate cross-contamination in a typical catalyst screening experiment with the AutoH^3^ system, we performed the styrene reduction sequentially through all six CCC port positions, alternating the cartridge between 30 mm 10% Pd/C CatCart^®^ cartridges (ports 1, 3, and 5) and 30 mm quartz CatCart^®^ cartridges (ports 2, 4, and 6). For each reaction, 1.0 mL of 0.5 M styrene in MeOH was injected and the reactions conducted at 30 °C, at a flow rate of 1.0 mL/min, in the full H_2_ mode. The sequence of reactions was conducted in order, from port 1 through to port 6 (Figure 6). The results clearly demonstrate that the active catalyst was leached from the 10% Pd/C CatCart^®^ cartridges and contaminated the subsequent experiments. The reaction sequence was repeated an additional six times, with the same cartridges in positions 1 through 6, for a total of 42 reactions in sequence (see Supporting Information File 1, Appendix B). At the end of the sequence of experiments, the conversion due to background contamination had reduced to 15%, which is still a significant value. The conversion due to background contamination was subsequently reduced to 0–5%, at 0.5 M concentration of styrene, after the system was washed by injections of acetic acid, dimethylformamide, and MeOH, in that order [21].

The results of these experiments prompted us also to examine the SAH^3^ system for residual system contamination. Prior to this examination, the SAH^3^ had been utilized for a variety of applications requiring the Pd, Pt, Rh and Raney Ni-containing CatCart^®^ cartridges. Two experiments were run with 2 mL injections of 0.5 M styrene in MeOH (30 mm quartz CatCart^®^, 1.0 mL/min, full H_2_ mode) resulting in 22.5% and 23.6% conversion due to background contamination (Table 2, entry 1). After washing with AcOH, DMF, and MeOH [22], the background reduction was reduced to 14.0–16.4% (Table 2, entry 2). To reduce the contamination further, the accessible parts of the system were removed and sonicated in 5 N HCl followed by MeOH. The stainless steel CatCart^®^ holder was contaminated with a dark residue that did not yield to cleaning by sonication or other mechanical means, so the holder was replaced. The Teflon membrane in the back-pressure regulator was replaced, as was the stainless steel mixing frit in the gas–liquid mixing chamber. After re-assembly and priming with MeOH, the level of conversion due to background contamination at 0.5 M styrene concentration was reduced to 2.2–3.2% (Table 2, entry 3), however, when the reaction was repeated at lower concentration (0.05 M styrene, manufacturer recommended), 6.1% and 11.5% conversion was observed (Table 2, entry 4).

**Table 2:** Background reduction of styrene to ethylbenzene caused by catalyst contamination on the SAH^3^ system.^a^

Entry	Details	[Styrene]	Conversion^b^

1	Prior to system wash	0.5 M	22.5, 23.6%
2	After system wash with AcOH, DMF, MeOH	0.5 M	14.6, 14.0, 16.4%
3	After system wash, disassembly and cleaning, test run 1	0.5 M	2.2, 3.0, 3.2%
4	After system wash, disassembly and cleaning, test run 2	0.05 M	6.1, 11.5%

^a^All reactions were performed in MeOH using a 30 mm quartz CatCart^®^ at a flow rate of 1.0 mL/min, full H_2_ mode, 30 °C, 2 mL injection per reaction, 10 mL volume collected. ^b^Conversion determined by GC–MS analysis with decane as an internal standard. Individual results from multiple experiments are shown.

The location of the remaining contamination was deduced through a series of bypass experiments (Table 3). The reduction was performed at 0.05 M styrene concentration with modifications to the reactor configuration as follows: (1) product was collected directly after passing through the CatCart^®^ by disconnection of the tubing from the outlet pressure sensor (Figure 1, G), and 0% conversion was observed (Table 3, entry 1); (2) tubing was re-routed directly from the CatCart^®^ holder to the back pressure regulator (Figure 1, H), thus bypassing the outlet pressure sensor, and resulting again in 0% conversion (Table 3, entry 2); finally, (3) tubing was disconnected from the back pressure regulator and product was collected after passing through the outlet pressure sensor, resulting in 5.5% and 8.8% conversions (Table 3, entry 3). Thus the remaining catalyst contamination was isolated to the outlet pressure sensor. Back-flushing the sensor with MeOH, 10% HNO_3_, water, and DMF failed to remove this contamination [23]. The problem was resolved by replacement of the sensor.

**Table 3:** Background reduction bypass experiments.^a^

Entry	Details	[Styrene]	Conversion^b^

1	Post-wash, back pressure regulator (H) and pressure sensor (G) bypassed	0.05 M	0%
2	Post-wash, pressure sensor (G) bypassed	0.05 M	0%
3	Post-wash, back pressure regulator (H) bypassed	0.05 M	5.5, 8.8%

^a^All reactions were performed in MeOH through a 30 mm quartz CatCart^®^ at a flow rate of 1.0 mL/min, full H_2_ mode, 30 °C, 2 mL injection per reaction, 10 mL volume collected. ^b^Conversion determined by GC–MS analysis with decane as an internal standard. Individual results from multiple experiments are shown.

**Figure 7:**
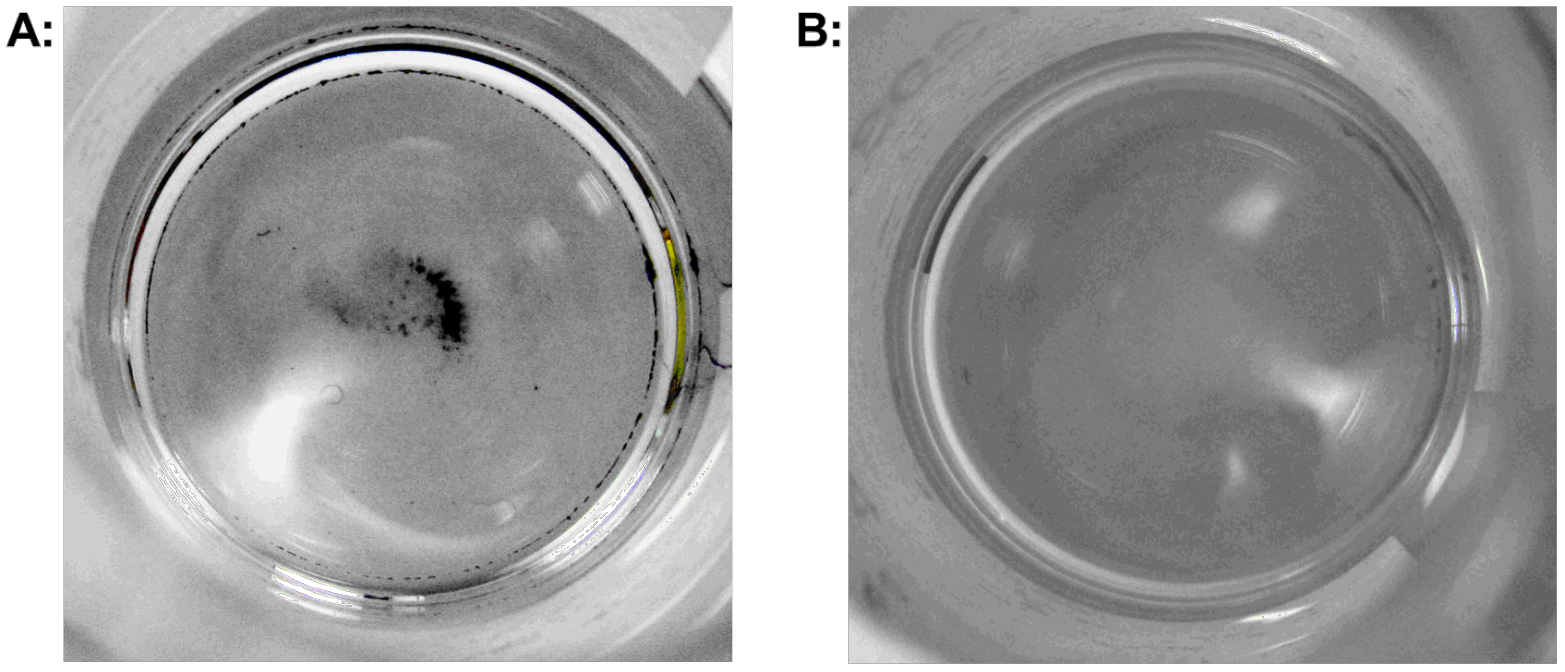
Leached catalyst from 30 mm 10% Pd/C CatCart^®^. First 10 mL wash aliquot (A) compared to second 10 mL wash aliquot (B). Photographs were taken from above, with the samples in 20 mL scintillation vials.

These results led us to suspect that solid catalyst particles were escaping from the catalyst cartridge and becoming trapped in the pressure sensor. To measure the amount of Pd washed from a CatCart^®^, we re-configured the SAH^3^ system to collect eluent directly downstream of the CatCart^®^ holder. A series of 10 mL aliquots of MeOH were collected sequentially from a previously unused 30 mm 10% Pd/C CatCart^®^ and the aliquots were analyzed for Pd content by ICP–MS. The washing process was conducted in the full H_2_ mode, at a flow rate of 2.0 mL/min, at 30 °C, to simulate a typical experiment. In the first 10 mL aliquot, 1.9 ppm of Pd was detected, corresponding to 19 μg Pd metal. In the subsequent three 10 mL aliquots, less than 1 ppm Pd was detected. When the wash samples were allowed to stand, a fine black free-flowing precipitate settled at the bottom of the first collection vial, whereas in the subsequent aliquot none was observed (Figure 7). It should be noted that Kappe observed a “Pd mirror” due to soluble Pd leaching from a CatCart^®^ during a continuous flow Mizorki–Heck reaction [24]. In comparison, the fine black precipitate seen in our wash experiment is more consistent with solid Pd/C catalyst. The experiment was repeated with a new 30 mm 10% Pd/C CatCart^®^ (from the same lot) to give 16 μg of Pd in the first 10 mL wash solvent. While this mass accounts for only ~0.1% of the average 150 mg catalyst loading per cartridge, the particle size is apparently very small and thus the relative catalytic activity is enhanced [25]. In addition, the contamination appears to accumulate through continued use of the reactor, presenting a signficant challenge with regard to performance stability. It should be noted that after thorough decontamination of the reactor we were able to reproduce the high chemoselectivity observed in the azide reduction in Scheme 1.

These observations, coupled with the previously discussed cartridge-to-cartridge performance variability, suggest that the best routine practice in handling commercial CatCart^®^ cartridges is to wash with MeOH first, followed by calibration against a known reaction, prior to use. We are currently evaluating the effectiveness of this protocol, in combination with the use of inline filters, on a larger sample of cartridges.

## Conclusion

We have shown that, for packed bed heterogeneous flow hydrogenation using the H-Cube^®^ reactor, (1) inconsistencies in the delivery of hydrogen in the controlled mode, (2) variable performance of the catalyst cartridges, and (3) system contamination can all result in significant variability of the reaction outcome, particularly at high substrate concentrations. It is important to reiterate that the manufacturer recommends a maximum working concentration of 0.1 M in order to mitigate the issues observed under hydrogen-limited conditions. Based on our improved understanding of the scope and limitations of the H-Cube^®^ reactor, we have applied the system to a variety of transformations and have achieved reproducible results. These results will be the subject of future reports.

## Experimental

### Instrument set up

**SAH^3^ reactor configuration.** A standard H-Cube^®^ hydrogenation flow reactor (ThalesNano Technology, Inc., Budapest, Hungary) was adapted to allow for fixed-loop injections and real-time monitoring by UV. The schematic is shown in Figure 1. The injector was a 6-port manual injector (Model C2-2006, Valco Instruments, Houston, TX) and the injection loop was obtained from Valco with a fixed volume of 2 mL. An LC-10A UV–vis detector (Shimadzu Corp., Columbia, MD) with a prep flow cell (0.1 mm path length) was connected downstream of the H-Cube^®^. Backpressure in the system was maintained at a minimum of 10 bar by an Upchurch back pressure regulator (Model M-410, Idex HS, Oak Harbor, WA) with an internal volume of 6 μL. Connection tubing between the HPLC pump and H-Cube^®^ was 0.020'' i.d. stainless steel with 1/16'' Valco HPLC fittings and lengths as short as possible to accommodate the unit in a standard bench top configuration. The tubing leaving the H-Cube^®^ was 0.007'' i.d. stainless steel (as part of the flow cell) and PEEK (as part of the Upchurch back pressure regulator).

**AutoH^3^ reactor configuration**. A standard H-Cube^®^ with CatCart Changer^®^ (ThalesNano Technology, Inc., Budapest, Hungary) was connected to a Gilson 215 liquid handler (Gilson Inc., Middleton, WI). The liquid handler bed was configured to hold 20 mL conical-bottom glass vials in a 14-vial Gilson rack (rack code 24) for sample injections. The bed was also configured to hold 14-vial Gilson racks containing 20 mL, septa capped, scintillation vials for product collection. The liquid handler was equipped with a 5 mL sample loop. Reaction sequences were programmed through the ThalesNano H-Cube^®^ auto sampler software.

### Reaction protocols

Solutions of styrene (ReagentPlus^®^, >99% purity) in MeOH (Chromasolv^®^, HPLC grade, >99.9%) were prepared in volumetric flasks, with 0.5 equiv anhydrous decane (>99% purity) included as an internal standard for quantification by GC. All reagents were purchased from Sigma-Aldrich and used as received. The CatCart^®^ cartridges were purchased from ThalesNano Technology, Inc. and, unless otherwise indicated, were washed with MeOH in the full H_2_ mode, at a flow rate of 1.0 mL/min, at 30 °C, for 10 minutes prior to use.

For reactions on the SAH^3^ system, the 2 mL injection loop was filled by injection of a 1.5 fold excess of the loop volume before each injection by a standard syringe with Luer-lock tip. During each filling, ~0.1 mL volume was left in the syringe to ensure that no air bubbles were introduced into the loop. Volumetric flow rate, system pressure, and temperature were controlled using the H-Cube^®^ front panel.

For reactions on the AutoH^3^ system, reagent solutions were charged to 20 mL conical vials and sealed with septa screw caps. Reaction conditions were programmed using the ThalesNano H-Cube^®^ sampler software. Unless otherwise noted, for each reaction a 1.9 mL injection was made and 10 mL product volume was collected in a 20 mL septa-capped scintillation vial.

### Analytical methods

The UV signal was monitored at a fixed wavelength of 265 nm with a LabJack U3 DAQ (Lakewood, CO) used to acquire the analog output from the in-line UV detector, at an acquisition rate of 20 Hz. Raw data was processed in Microsoft Excel and SigmaPlot (Systat Software, San Jose, CA).

Product mixtures were analyzed by GC–MS with a Hewlett Packard HP 6890 series GC equipped with a CTC analytics-MSPAL auto injector and a HP 5973 mass selective detector [transmission quadrupole mass spectrometer, electron ionization (EI)]. An Agilent J&W DB-XLB 30 m × 0.25 mm i.d. capillary column, with a 0.5 micron film, was used in combination with the following oven temperature program: An initial temperature of 70 °C held for 5.0 min, then a 50 °C/min ramp to the final temperature of 200 °C, held for 1.0 min. The injector temperature was set to 250 °C and the ion source temperature was set to 230 °C. Helium (grade 5.0 purity, 100%) was used as the carrier gas with a gas flow of 37 cm/s average linear velocity and at a pressure of 8.90 psi. Either a split method (50:1 split ratio, 50 mL/min split flow) or a splitless method was used, depending upon sample concentration. Samples were diluted to 0.04 M when the split method was used, or to 0.004 M when the splitless method was used. Mass data was acquired after a 3.00 min solvent delay, and scan parameters covered the range of 15.0–550.0 amu. Data analysis was performed using Agilent GC/MSD Chemstation software.

## Supporting Information

Supporting information features experimental queues for which an “instability detected” status was recorded by the auto sampler software during the run, and a full data set for the alternating Pd/quartz experiment.

File 1 Experimental queues and alternating Pd/quartz results.
