# Supplementary material for: Evaluation of a commercial packed bed flow hydrogenator for reaction screening, optimization, and synthesis
Source: Beilstein J Org Chem. 2011 Aug 22;7:1141–9. doi: 10.3762/bjoc.7.132 (PMC3170201; doi:10.3762/bjoc.7.132)
Supplement: File 1 — Experimental queues and alternating Pd/quartz results. [file Beilstein_J_Org_Chem-07-1141-s001.pdf]

Supporting Information

for

Evaluation of a commercial packed bed flow hydrogenator for reaction screening, optimization, and synthesis

Marian C. Bryan[1], David Wernick[2], Christopher D. Hein[1],

James V. Petersen[3], John W. Eschelbach[2] and Elizabeth M. Doherty[1]*

[1]Medicinal Chemistry Research Technology, Department of Chemistry Research and Discovery, Amgen, Inc., One Amgen Center Drive, Thousand Oaks, CA 91320, USA, [2]Discovery Analytical Sciences, Department of Chemistry Research and Discovery, Amgen, Inc., One Amgen Center Drive, Thousand Oaks, CA 91320, USA and [3]Research Automation Technology, Department of Chemistry Research and Discovery, Amgen, Inc., One Amgen Center Drive, Thousand Oaks, CA 91320, USA

Email: Elizabeth M. Doherty - edoherty@amgen.com

* *Corresponding author*

Experimental queues and alternating Pd/quartz results

Evaluation of a Commercial Packed Bed Flow Hydrogenator for Reaction Screening, Optimization, and Synthesis

Supplemental Information

Appendix A

Experimental queues for which an "instability detected" status was recorded by the auto sampler software during the run.

Entry	Queue date	Experiment # in queue	H2 mode	Flow rate (mL/min)	Temperature (°C)	Pressure setting (bar)	Status
1	6/7/2010	1	full	1	60		Collected
		2	controlled	1	60	60	Collected with Error (instability detected)
		3	controlled	1	60	60	Collected
2	6/25/2010	1	controlled	0.5	60	60	Collected with Error (instability detected)
		2	controlled	1	60	60	Collected
3	9/22/2010	1	full	1	30		Collected
		2	full	1	30		Collected
		3	full	1	30		Collected
		4	controlled	1	30	100	Collected with Error (instability detected)
4	9/22/2010	1	full	1	30		Collected
		2	full	1	30		Collected
		3	full	1	30		Collected
		4	full	1	30		Collected
		5	controlled	1	30	10	Collected
		6	controlled	1	30	10	Collected
		7	controlled	1	30	10	Collected
		8	controlled	1	30	10	Collected
		9	controlled	1	30	10	Collected
		10	controlled	1	30	30	Collected
		11	controlled	1	30	30	Collected
		12	controlled	1	30	30	Collected
		13	controlled	1	30	30	Collected
		14	controlled	1	30	30	Collected
		15	controlled	1	30	60	Collected
		16	controlled	1	30	60	Collected
		17	controlled	1	30	60	Collected
		18	controlled	1	30	60	Collected
		19	controlled	1	30	60	Collected
		20	controlled	1	30	80	Collected with Error (instability detected)
		21	controlled	1	30	80	Collected
		22	controlled	1	30	80	Collected
		23	controlled	1	30	80	Collected
		24	controlled	1	30	80	Collected
		25	controlled	1	30	100	Collected
		26	controlled	1	30	100	Collected
		27	controlled	1	30	100	Collected
		28	controlled	1	30	100	Collected
5	9/27/2010	1	controlled	1	30	10	Collected
		2	controlled	1	30	10	Collected
		3	controlled	1	30	10	Collected
		4	controlled	1	30	80	Collected with Error (instability detected)
		5	controlled	1	30	80	Collected
		6	controlled	1	30	80	Collected with Error (instability detected)
6	9/27/2010	1	controlled	1	30	10	Collected with Error (instability detected)
		2	controlled	1	30	10	Collected
		3	controlled	1	30	80	Collected with Error (instability detected)
		4	controlled	1	30	80	Collected with Error (instability detected)
7	4/15/2011	1	full	1	30		Collected
		2	controlled	1	30	60	Collected with Error (instability detected)
		3	controlled	1	30	60	Collected
		4	controlled	1	30	60	Collected

Appendix B

Full data set for conversion of styrene to ethylbenzene over sequential reactions alternating 10% Pd/C with quartz on the AutoH^3 system.[a]

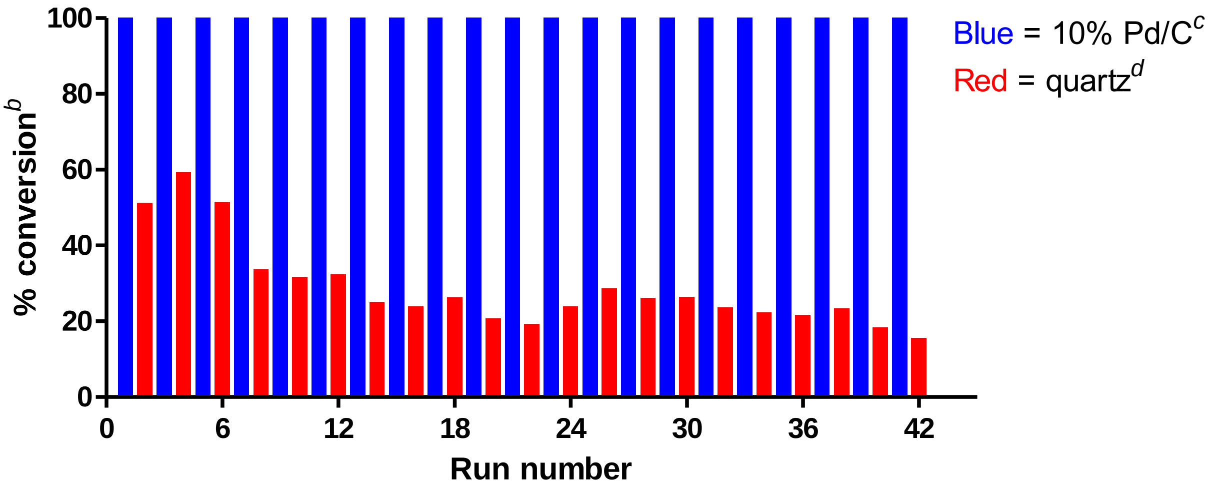

[a]All reactions were performed at a flow rate of 1.0 mL/min, temperature 30 °C, 0.5 M styrene in MeOH, 1 mL injection per reaction, run in order 1 through 6, then repeated in order 1 through 6. [b]Conversion based on GC-MS analysis using decane as an internal standard. [c]Data represented in blue correspond to reactions performed through a 30 mm 10% Pd/C CatCart™. [d]Data represented in red correspond to reactions performed through 30 mm quartz CatCart™.
